# Supplementary material for: All-optically controlled phased-array for ultrasonics
Source: Nat Commun. 2025 Aug 29;16:8091. doi: 10.1038/s41467-025-63517-9 (PMC12397371; doi:10.1038/s41467-025-63517-9)
Supplement: Supplementary file 1 — Supplementary Information [file 41467_2025_63517_MOESM1_ESM.pdf]

Supplementary Information:
All-Optically Controlled Phased-Array for Ultrasonics

Rahul Goyal, Oscar Demeulenaere, Marc Fournelle,
Athanasios G. Athanassiadis, Peer Fischer

CONTENTS

Supplementary Note 1: Analysis of the optically-active phase shift circuit **2**

Supplementary Note 2: Electrical characteristics of the transducer element **3**

Supplementary Note 3: Electronics board design and implementation **4**

Supplementary Note 4: Optical and electrical characteristics of light addressable phase-shifter **5**

Supplementary Note 5: Measurement of Ultrasound Wavefronts **6**

Supplementary Note 6: Measurement of phase uniformity in emitted ultrasound wavefront **7**

Supplementary Note 7: Performance of the circuit for broadband pulses **8**

Supplementary Figure 1: Parallel electrical connections for the operation of board **9**

Supplementary Figure 2: Simulation of the light-addressable phase shifter **10**

Supplementary Figure 3: Dependence of phase shift on transducer capacitance **11**

Supplementary Figure 4: Impedance analysis of loaded piezoelectric element operated at 2.25 MHz **12**

Supplementary Figure 5: Impedance analysis of loaded piezoelectric element operated at 0.7 MHz **13**

Supplementary Figure 6: Impedance measurements of transducer elements operated at 0.7 MHz **14**

Supplementary Figure 7: Photographs of the experimental system (OPAT) **15**

Supplementary Figure 8: Snapshots of the electronics board **16**

Supplementary Figure 9: Optical active area in the light-addressable phase shifter **17**

Supplementary Figure 10: Photographs of the acoustic scanning system to map the acoustic pressure **18**

Supplementary Figure 11: Experimental setups for acoustic pressure measurements **19**

Supplementary Figure 12: Switching of ultrasound phase at 100 Hz **20**

Supplementary Figure 13: Phase uniformity in the emitted ultrasound wavefront **21**

Supplementary Figure 14: Broadband response of light-addressable transducer array **22**

Supplementary Note 1

Analysis of the optically-active phase shift circuit

The phase shift circuit utilizes resistors and capacitors in an arrangement as shown in Fig. **S1** (A) or Fig 2 (A). The transducer is modeled as an element with complex impedance, Z_T. The effective voltage V_T applied to the transducer element can be written as,

$$V_T = V_i \times \left[\frac{Z_T[(R(R+2X_{C_S}) + X_{C_P}(X_{C_S} - R)]}{(R+2X_{C_S})(Z_T X_{C_P} + R(Z_T + X_{C_P})) - X_{C_P} X_{C_S} Z_T} \right] \tag{S1}$$

where,

- X_{C_S} is the reactance of the series capacitance C_S
- X_{C_P} is the reactance of the parallel capacitance C_P
- R is the optically controllable resistance, and
- V_i is the input voltage signal.

The complex impedance of transducer element Z_T can be expressed as,

$$Z_T = R_T + X_T = R + 1/(j\omega C_T), \tag{S2}$$

similarly, X_{C_S} and X_{C_P} are $1/(j\omega C_S)$ and $1/(j\omega C_P)$, respectively. The input signal V_i is sinusoidal and has a peak voltage of V_P and the frequency F ($\omega = 2\pi F$). Substitution of X_{C_S}, X_{C_P}, and Z_T in equation (S1) yields the effective voltage V_T (written in the complex form),

$$V_T = V_i \times \left[\frac{\begin{array}{c}\omega(R_T C_T - \omega^2 R^2 R_T C_S C_P C_T - R C_S + 2R C_P) \\ - j(\omega^2 R R_T (C_S C_T - 2C_P C_T) + 1 - \omega^2 R^2 C_S C_P)\end{array}}{\begin{array}{c}-\omega R(C_S + 2C_P + 2C_T) - \omega R_T C_T + \omega^3 R^2 R_T C_S C_P C_T \\ - j(\omega^2 R R_T C_T (C_S + 2C_P) + \omega^2 R^2 C_S (C_P + C_T) - 1^{)}\end{array}} \right] \tag{S3}$$

Therefore, the magnitude and phase of the effective output voltage V_T can be expressed as

$$V_T = V_i \times \left[\frac{\sqrt{\begin{array}{c}(\omega(R_T C_T - \omega^2 R^2 R_T C_S C_P C_T - R C_S + 2R C_P))^2 + \\ (\omega^2 R R_T (C_S C_T - 2C_P C_T) + 1 - \omega^2 R^2 C_S C_P)^2\end{array}}}{\sqrt{\begin{array}{c}(\omega R(C_S + 2C_P + 2C_T) - \omega R_T C_T + \omega^3 R^2 R_T C_S C_P C_T)^2 + \\ (\omega^2 R R_T C_T (C_S + 2C_P) + \omega^2 R^2 C_S (C_P + C_T) - 1^{)^2}\end{array}}} \right] \tag{S4}$$

and

$$\angle V_T = \tan^{-1}\left[\frac{-(\omega^2 R R_T (C_S C_T - 2C_P C_T) + 1 - \omega^2 R^2 C_S C_P)}{\omega(R_T C_T - \omega^2 R^2 R_T C_S C_P C_T - R C_S + 2R C_P)} \right] - \tan^{-1}\left[\frac{-(\omega^2 R R_T C_T (C_S + 2C_P) + \omega^2 R^2 C_S (C_P + C_T) - 1)}{-\omega R(C_S + 2C_P + 2C_T) - \omega R_T C_T + \omega^3 R^2 R_T C_S C_P C_T} \right], \tag{S5}$$

respectively. According to equation (S5), by changing the resistance R of the dual-cascaded balanced RC circuit, the phase of V_T at the operating frequency will change accordingly, i.e., the light intensity controls the resistance R of the drive network which in turn directly shifts the phase of the ultrasound wave. A transducer element with a large Q factor can realize a $-\pi$ to $+\pi$ phase shift over a wide frequency range. Further, according to equation (S5), it follows that, the phase ($\angle V_T$) is approximately independent of the equivalent capacitance of the actuator element (C_T). The phase-shift ($\Delta\phi = \angle V_T - \angle V_i$) as a function of resistance (R) is plotted in Fig. **S2** (A). The net $\Delta\phi$ with R is plotted for different capacitance values ($C_T = \{100\,\text{pF}, 200\,\text{pF}, 300\,\text{pF}, 400\,\text{pF}, 500\,\text{pF}\}$) and it is seen that the the phase-shift is independent of the capacitance of the transducer element. Similarly, the net phase-shift, $\Delta\phi_T$ (max($\Delta\phi$) - min($\Delta\phi$)) as a function of frequency (F) is represented graphically in the Fig. **S2** (B) for various different capacitance values. The net phase-shift, $\Delta\phi_T$ (max($\Delta\phi$) - min($\Delta\phi$)) at an operating frequency (F) of 0.7 MHz as a function of capacitance of the transducer element (C_T) is calculated and plotted as shown in Fig. **S3**. Thus, even if the capacitance of transducers operating at different frequencies changes, the phase control is stable across a wide operating bandwidth for different load capacitances.

Supplementary Note 2

Electrical characteristics of the transducer element

We performed electrical impedance measurement of the transducer element, using a 5 MHz lock-in amplifier equipped with a dedicated impedance analyzer (Zurich Instruments) . We measured the characteristics of both transducers used to evaluate the OPATS: a 2D array with elements resonant at 0.7 MHz and a single element disk resonant at 2.25 MHz. Since the $R_S + C_S$ model is the most generalized model to explain the electrical impedance of transducer elements as shown in Fig. **S4** (A) [1, 2], we used the built-in $R+C$ model in the Zurich Instruments LabOne software and obtained values for R_T and C_T for each transducer as a function of frequency.

The results for the 2.25 MHz lead zirconate titanate (PZT) piezoelectric disk are shown in Fig. **S4**. The impedance is measured across the frequency range 100 kHz to 5 MHz. The equivalent resistance and capacitance of the transducer are plotted in Figs. **S4** (B) and (C). The values of R_T and C_T at the frequency of 2.25 MHz are $\approx 380\,\Omega$ and $\approx 75\,\text{pF}$, respectively. The magnitude of the resistive component (R_T), and the reactive component (X_T) of the complex impedance (Z_T) are shown in Fig. **S4** (D). It is seen from Fig. **S4** (D) that the magnitude of complex impedance ($|Z_T|$), which is given by,

$$|Z_T| = \sqrt{R_T^2 + X_T^2} \tag{S6}$$

is dominated by the capacitance. Moreover, to confirm that the phase shifting performance primarily depends on the capacitive loading of the transducer, we numerically simulate the OPAT phase shifting performance as a function of R_T with a fixed capacitance $C_T = 75\,\text{pF}$ for operation at 2.25 MHz. As shown in Fig. **S4** (E), the phase shifting performance is effectively independent of transducer resistive loading.

The electrical characterization of one element of the 0.7 MHz array is shown in Fig. **S5** and Fig. **S1** (B). The experimental results support the equivalent of substitution the transducer element as a capacitor. We have characterized the individual elements of the 11×11 transducer array and the results are shown in Fig. **S6**. The capacitive component C_T for every element is plotted in Fig. **S6** (A), where Fig. **S6** (C) shows the distribution of the capacitances at 0.7 MHz. Similarly, the element-wise distribution of the resistive component (R_T) is shown in Fig. **S6** (B) and (D).

Supplementary Note 3

Electronics board design and implementation

The optically controlled transducer array is implemented by projecting light from commercially available light projector (ViewSonic PRO8510L-S Light Stream 5200-Lumen Projector) to a 2D array of photoresistors (NSL-19M51, Advanced Photonix). The overall device including the projector, the printed circuit board and the 11x11 array transducer is shown in Fig. **S7**.

The printed circuit board for the array of photoresistors is shown in Fig. **S8** (A). The board has dimensions of 351 mm × 285 mm and a thickness of 1.55 mm. As seen in Fig. **S8** (A) a total of $2 \times 11 \times 11$ photoresistors are mounted in close proximity to decrease the net footprint of the array. The board has a Samtec Socket Strip with a pitch of 2.54 mm which is designed to be connected with pin headers of identical pitch and hole dimensions.

The photoresistor board is connected to the capacitor board as is shown in Fig. **S8** (B). The capacitor board performs the light intensity-dependent electrical phase-shift via a cascaded architecture. The capacitor board is mounted with through-hole pin headers directly with the socket strip of the photoresistor board. The pin headers are rated for operation up to 3.2 A with a high-speed channel bandwidth of 8 Gbps, which provides 12-bit resolution to the electrical signal of 1 MHz. The PCBs are designed in the computer-aided software KiCad 6.1 using components from standard libraries. The capacitors of dimension code 0603 are surface mounted on the capacitor board as shown in Fig. **S8** (B). The capacitors are thin film ceramic capacitors with a maximum operational voltage of 250 V.

The input power is supplied through a BNC coaxial connector and it should be noted that the input power connectors are central to the individual elements of the transducer array which does not depend on the number of elements in the array. The placement of the ITT canon connector on the capacitor board is shown in Fig. **S8** (B). The capacitor board is designed with a six layer architecture. The PCBs are made by Eurocircuits Gmbh and the components were installed in our laboratory.

Supplementary Note 4

Optical and electrical characteristics of light addressable phase-shifter

We account for the energy consumption of our OPAT architecture by considering (1) the optical power necessary for phase shifting, and (2) the additional electrical power requirements of the phase-shifting circuits.

The optical power consumed by the light addressable phase-shifter circuit depends on the photoresistors used and the light transmission geometry. Following the schematic shown in Fig. **S9** (A) we consider the energy required to illuminate the total bounding box of the photoresistor array. The total illuminated area $T_A = L_A \times W_A$, where $L_A = 77.5\,\text{mm}$ and $W_A = 55.1\,\text{mm}$ for our implementation, resulting in a total illumination area of $T_A \approx 4270\,\text{mm}^2$. This area is used to control 121 elements of the transducer array, as shown in Fig. **S9** (A) and Fig. **S9** (B).

The individual light addressable phase unit comprises two photoresistor which should be identically illuminated. We measured the incident net light intensity corresponding to the maximum illumination level in the experimental conditions, to be $I_{MAX} = 65.7\,\text{mW/cm}^2$. The maximum optical power consumed to operate the phase control per pixel is therefore $P_{optical} = I_{MAX} \times T_A \approx 23\,\text{mW}$. The use of more sophisticated light projection systems can reduce the required optical power, as can the use of more sensitive optically active elements in the circuit.

We assessed the electrical power draw of the OPAT by measuring the electrical power flow before and after the OPAT circuitry while driving the 11×11 array. Power flow is measured using an inline 20 dB bidirectional coupler (Minicircuits ZFBDC20-61HP) with the forward and reverse signals read by a USB oscilloscope (Picoscope 5440). This configuration allowed us to fully resolve the transmitted 25-cycle waveforms. The RMS power is then calculated from the measured voltage and circuit impedance ($R = 50\,\Omega$) during the pulse ($P = V_{RMS}^2/R$). In the dark state ($\Delta\phi = -\pi$) we measured an RMS power of 3.6 W flowing into the OPAT and transducer during a single pulse, corresponding to a power of 30 mW per pixel. In the light state ($\Delta\phi = +\pi$) the RMS power draw was 2.7 W, corresponding to a power draw of 23 mW per pixel. The differences in the two states arise because the circuit impedance changes with the photoresistor's values. Since in practice, each pixel of the array will be assigned a different phase shift and therefore a different light intensity, we can use these two cases to bound the power consumption of the array driven by the OPAT.

In order to separate the useful acoustic power transmitted into the circuit from the parasitic electrical losses within the circuit itself, we then measured the power flowing from the OPAT into a single pixel of the array and subtracted this from the total electrical power flowing into the OPAT. We measured transducer powers of 27 mW and 17 mW in the dark and light states, respectively. This corresponds to a bound on the parasitic losses between 3 mW and 6 mW per OPAT pixel.

In our current implementation, therefore, optical power consumption to induce the photoresistive phase shift dominate the overall power consumption, which could be the focus of initial efforts to improve the power efficiency. This could be achieved, for instance, by optimizing the light projection and collection hardware using a micro-LED array coupled to a microlens array to effectively couple all of the transmitted light onto the photosensitive elements.

Supplementary Note 5

Measurement of Ultrasound Wavefronts

The ultrasound pressure field is mapped by hydrophone scanning in a plane. An open 3D printed water tank is filled with deionized water. The pressure waves transmit through the water and a needle hydrophone (HNR0500, 0.5mm in diameter Onda Corporation) is used to record the amplitude and phase of the ultrasound wavefront. The hydrophone is mounted on a three-axis motorized translation stage (MTS25-Z8, Thorlabs GmbH) to measure the ultrasound wavefront across the imaging plane. A photograph of the setup is shown in Fig. **S10**.

Several acoustic scans were performed depending on the experiment. The experimental setups are illustrated in Fig. **S11**.

- Determination of phase patterns near the plane of the transducers
- Focus and multi-focus measurements
- Temporal response during switching between 2 foci.

The switching speed of the cascaded network restrains the refresh rate of the signal modulation. This refresh rate determines the frame rate at which the ultrasound wavefront can be updated through light intensity patterns. The response times of the electrical cascaded network theoretically allows a maximum update rate of 100 Hz. The optical switching of the ultrasound phase from $-\pi$ to $+\pi$ and vice versa at 100 Hz is demonstrated experimentally in Fig. **S12**.

Supplementary Note 6

Measurement of phase uniformity in emitted ultrasound wavefront

The uniformity in the phase of emitted ultrasound wavefront is measured to characterize the phase controllability using light. The total acoustic aperture of 30.9 mm $\times$ 30.9 mm is scanned to determine the phase information from wavefront. The near field measurement is performed with a hydrophone placed 2.0 mm from the transducer face (see Fig. **S13** (A) for schematic representation of the setup). We projected square-shaped patterns with different light intensities as shown in Fig. **S13** (B) (i) - (V) onto the LAPS. The dimension of the projected pattern is such that we control the phase of transducer elements in a 5×5 sub-array. The associated phase-shifted driving signals produced the spatially-varying pressure fields, which we scanned using a hydrophone. The resulting pressure amplitudes and phases defining the emitted wavefronts are shown in Figs. **S13** (C) and (D), respectively. The projected pressure fields consist of constant ultrasound amplitudes with the only small deviations observable near sharp phase boundaries, as expected. The phase on the other hand is progressively shifted by $\pi/3$ in accordance with the increased light intensity incident on the OPAT. The performance of a single LAPS scales well to an entire array, providing well-defined, controllable phase shifts across the entire acoustic wavefront. The mean and standard deviation of the phase corresponding to the 5×5 sub-array of transducer elements are plotted in Fig. **S13** (E). It is seen that the phase-shift of the ultrasound waves is in accordance with the corresponding change in the light intensity. The variability in the phase can be seen to arise from diffraction of the wave across the sharp boundaries, leading to interference within the uniform square by the time the pressure wave reaches the hydrophone.

Supplementary Note 7

Performance of the circuit for broadband pulses

We assessed the performance of the circuit for broadband pulses by transmitting a single-cycle sine pulse at 0.7 MHz through the ultrasound array both with and without the phase shifting circuit. The time signals of the drive signal and of the measured pressure signals are plotted in Fig. **S14** (A). After FFT, the amplitude (B) and phase (C) are plotted over a frequency range up to 1.5 MHz. By plotting the phase difference between the output pressure wave with and without the circuit in (D), one can observe that the circuit can apply a nearly constant phase shift across the operating band 0.2 MHz – 1.2 MHz. Therefore, the OPAT can be used to transmit and apply a constant phase shift to broadband pulses with energy concentrated in this band.

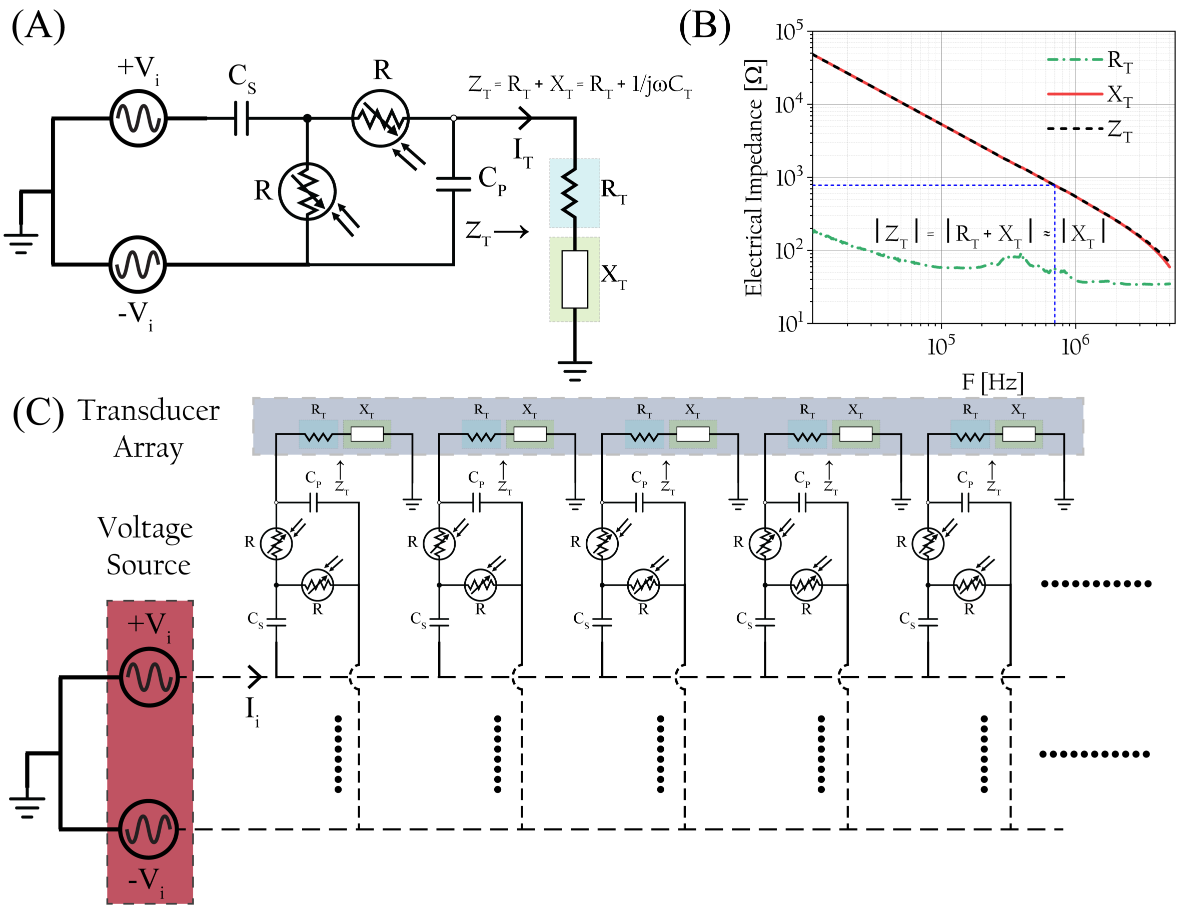

Supplementary Figure S1: **Parallel electrical connections for the operation of board.** (A) Simplified circuit diagram of the programmable pixel unit where the loaded transducer element is modeled as a passive element with complex electrical impedance (Z_T). (B) Measured complex electrical impedance (Z_T) with resistive (R_T) and reactive (X_T) component as a function of frequency. (C) Electrical circuit diagram of the Optically Programmable Array of Transducers to control the phase of emitted ultrasound waves from transducer elements.

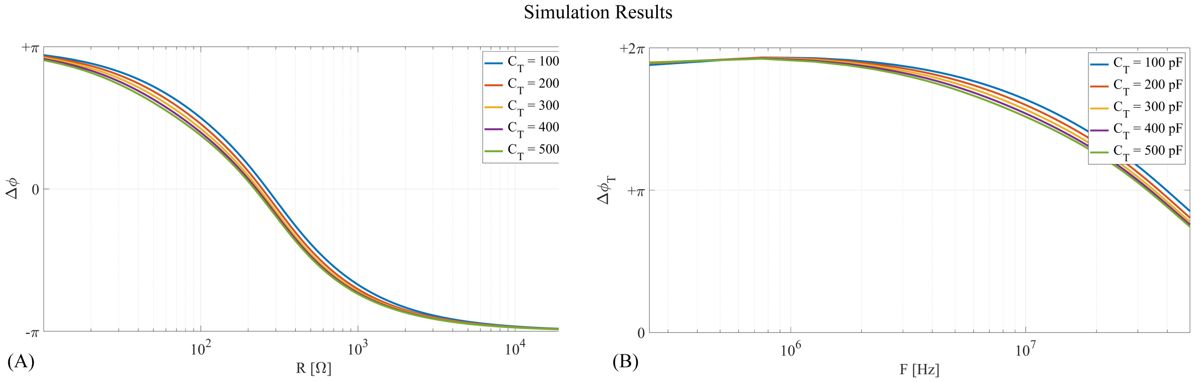

Supplementary Figure S2: **Simulation of the light-addressable phase shifter.** (A) Variation of phase-shift, $\Delta\phi$ for different values of transducer capacitance (C_T) as a function of Resistance (R) of photoactive components. (B) Variation of the net phase-shift, $\Delta\phi_T$ (max($\Delta\phi$) - min($\Delta\phi$)) for different values of transducer capacitance (C_T) as a function of operation frequency (F).

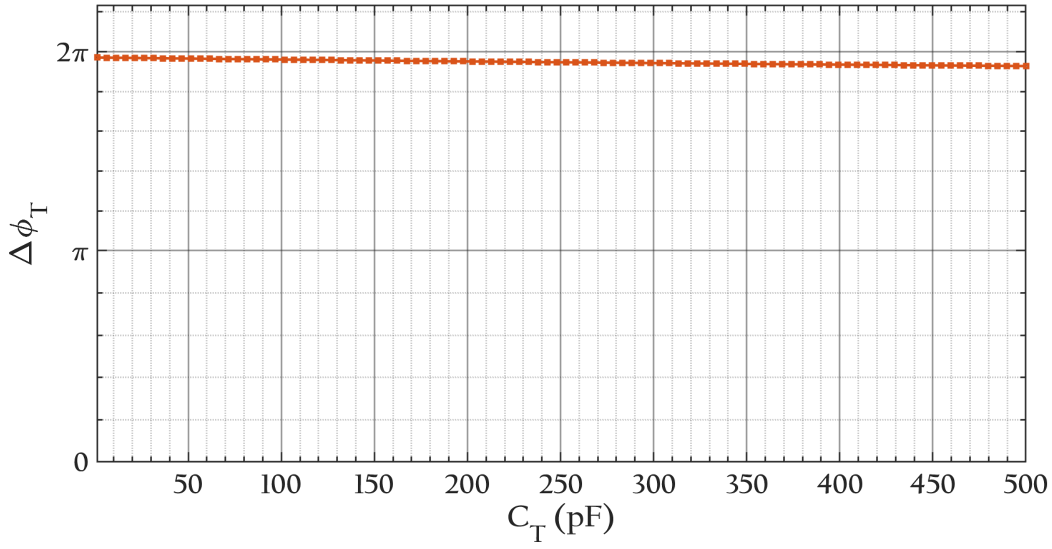

Supplementary Figure S3: **Dependence of phase shift on transducer capacitance.** Variation of the net phase-shift, $\Delta\phi_T$ (max($\Delta\phi$) - min($\Delta\phi$)) for different values of transducer capacitance (C_T).

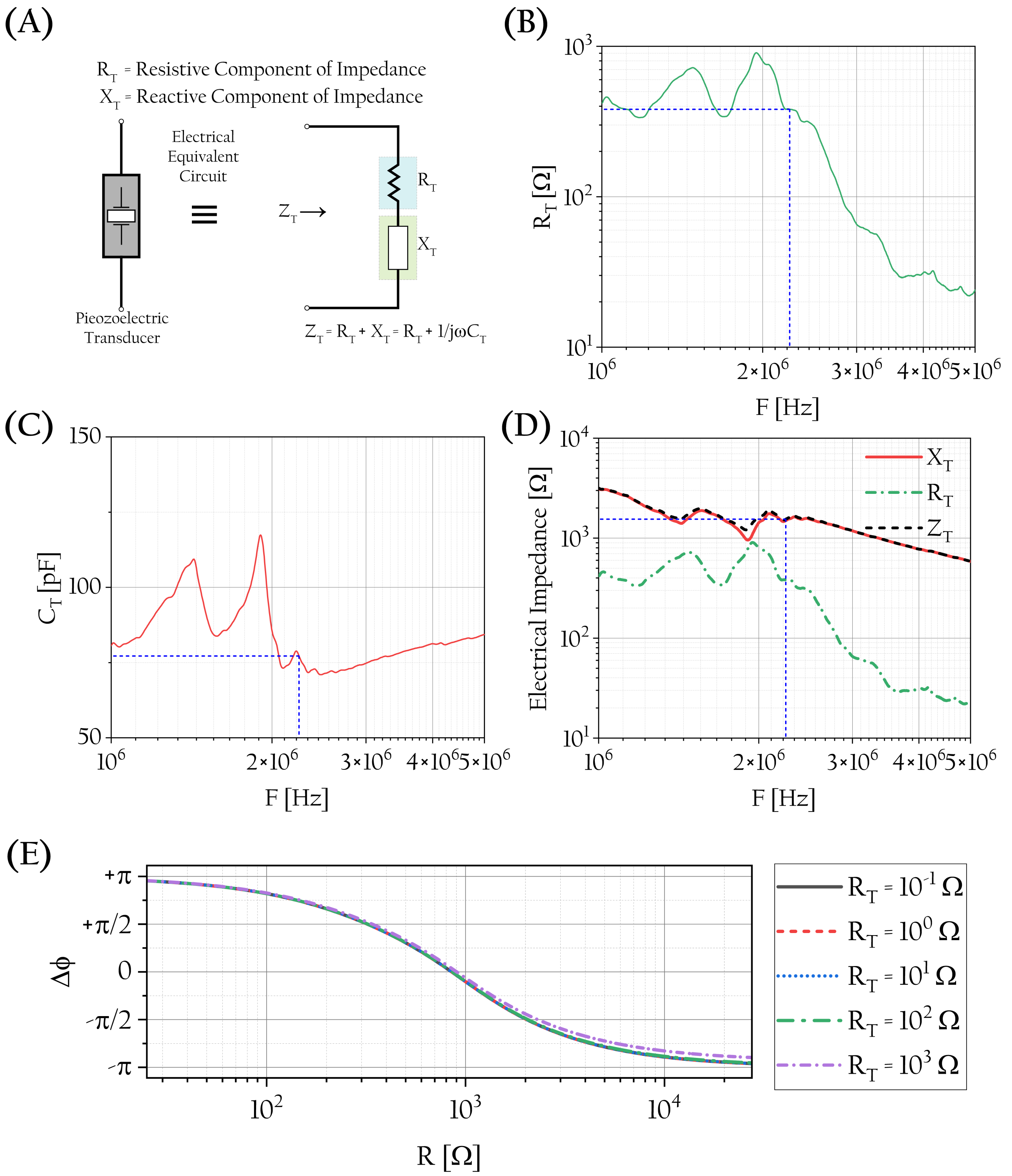

Supplementary Figure S4: **Impedance analysis of loaded piezoelectric element operated at** 2.25 MHz**.** (A) Generalized equivalent electrical model for the piezoelectric element. (B) Measured resistive component (R_T) of the complex impedance (Z_T) as a function of frequency. (C) Measured capacitance as a function of frequency. (D) Measured complex electrical impedance (Z_T) with resistive (R_T) and reactive (X_T) component as a function of frequency. (E) Calculated phase shift as a function of photoresistor resistance. The phase shift is effectively independent of the resistive loading from the transducer R_T.

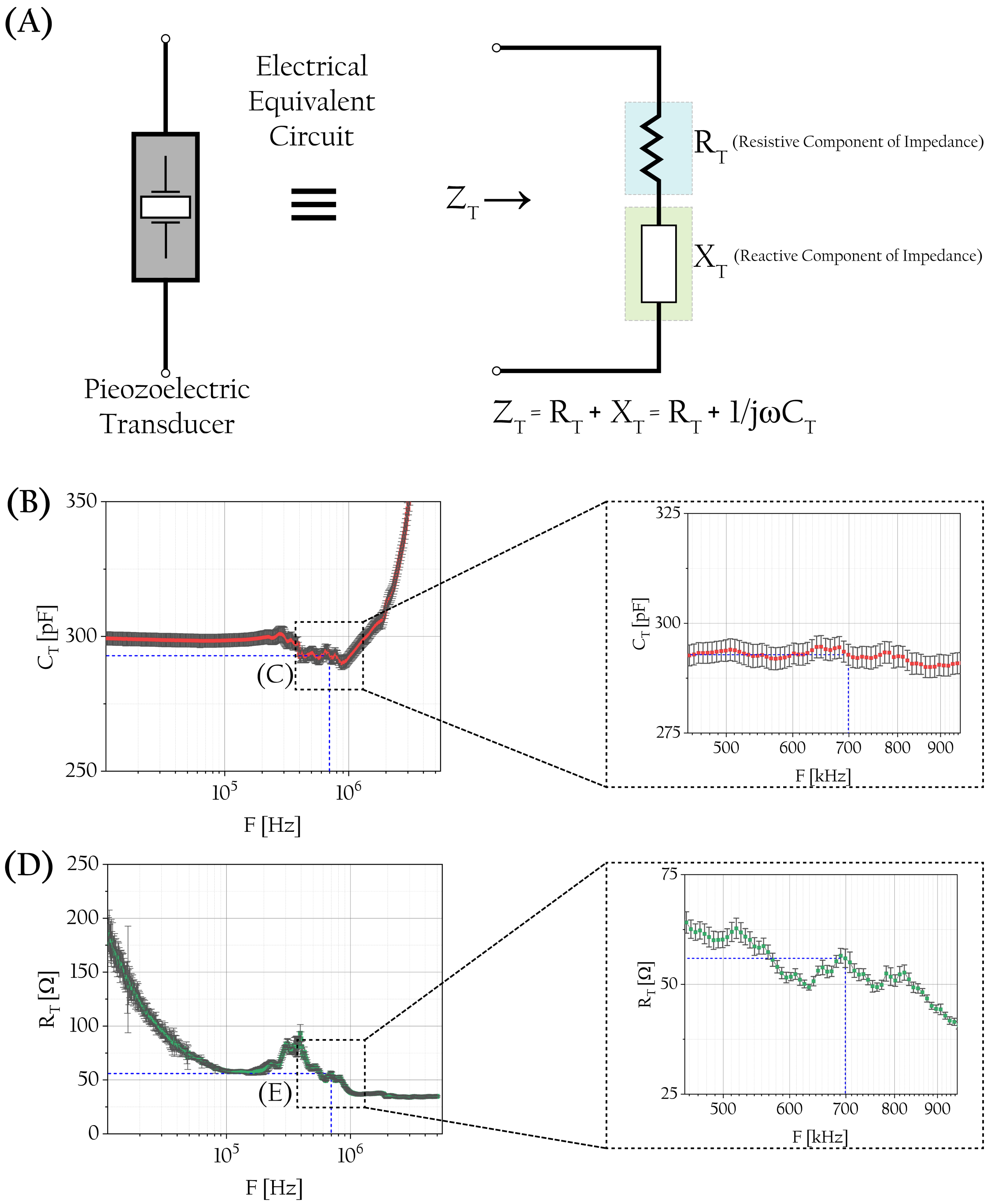

Supplementary Figure S5: **Impedance analysis of loaded piezoelectric element operated at** 0.7 MHz**.** (A) Generalized equivalent electrical model for the piezoelectric element. (B) Measured capacitance as a function of frequency. (C) The inset shows the value of capacitance (C_T) at operational frequency of 0.7 MHz. Data is presented as mean value ± standard deviation in the mean value of the capacitance. (D) Measured resistive component (R_T) of the complex impedance (Z_T) as a function of frequency. (E) The inset shows the value of resistance (R_T) at operational frequency of 0.7 MHz. Data is presented as mean value ± standard deviation in the mean value of the resistance.

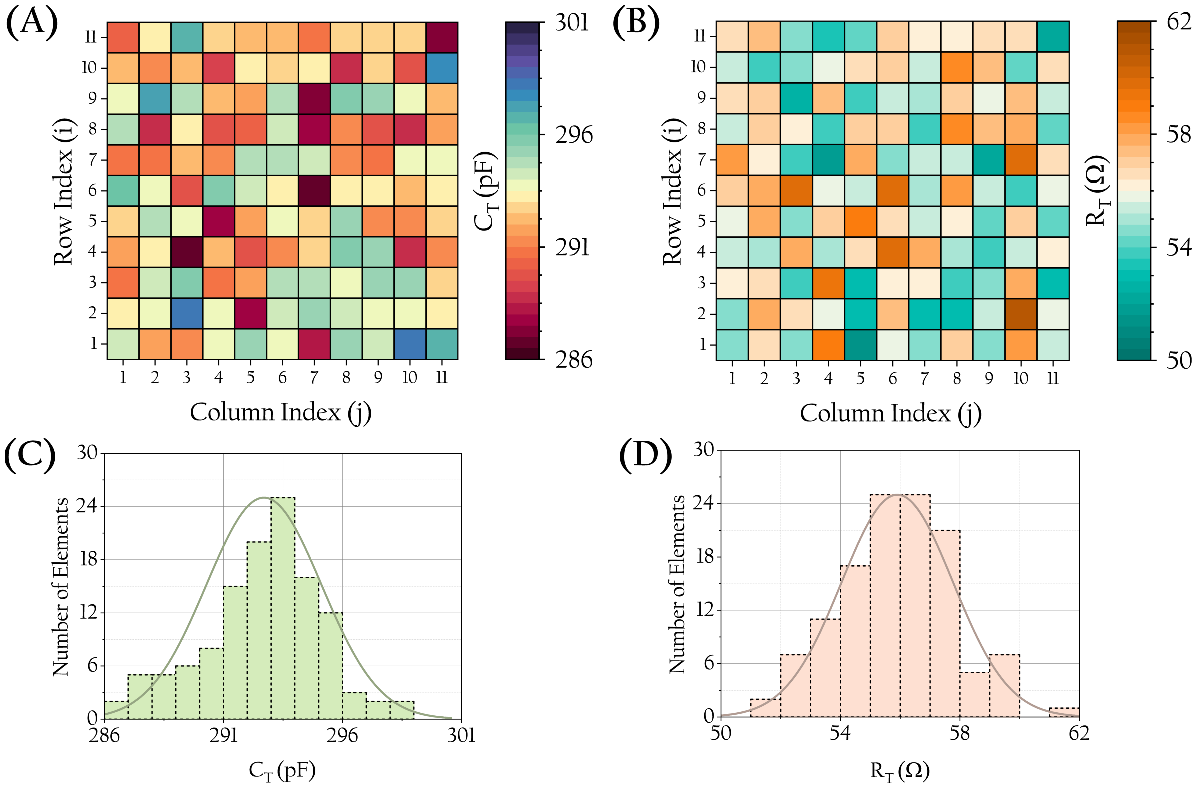

Supplementary Figure S6: **Impedance measurements of transducer elements operated at 0.7 MHz.** Surface distribution of the (A) capacitance and (B) resistance values for the 121 elements of the transducer array. Histogram representation of the (C) capacitance and (D) resistance values for the 121 elements of the transducer array.

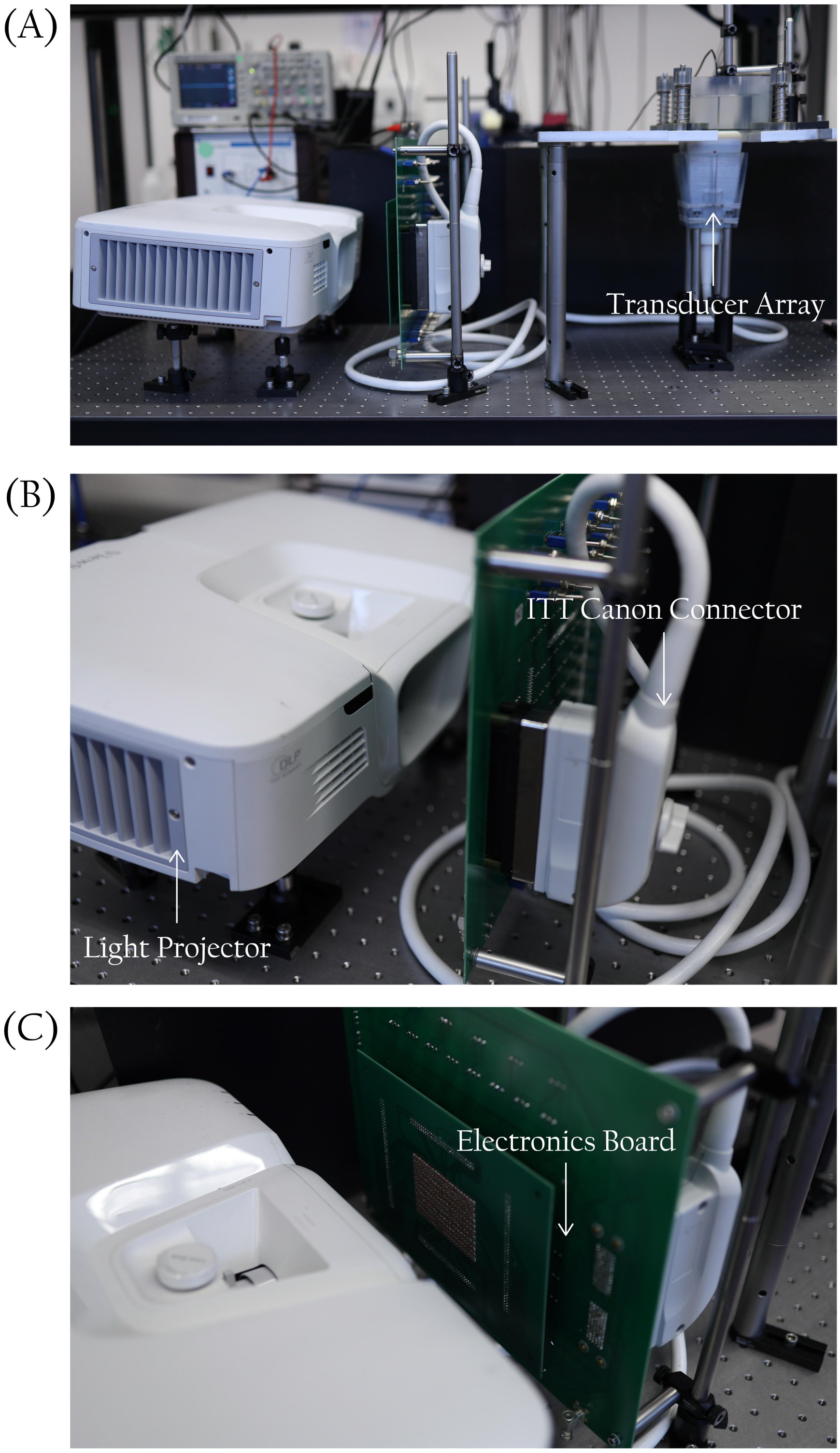

Supplementary Figure S7: **Photographs of the experimental system (OPAT).** The experimental system includes, the light projector, optically-addressable electronics board, and transducer array. (A) Front view. (B) Perspective view. (C) Top view.

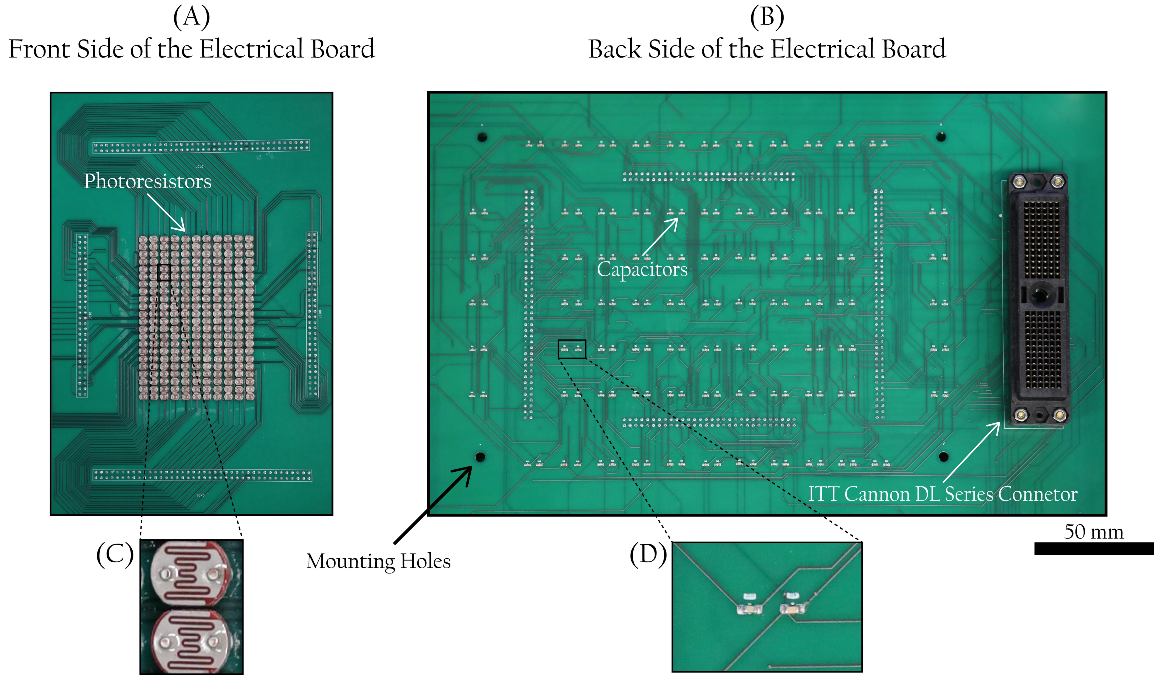

Supplementary Figure S8: **Snapshots of the electronics board.** (A) Design of the printed circuit board for the photoresistor panel of the OPATs where the white arrow shows the photoresistors mounted on the front side of the board. (B) The back side of the printed circuit board containing the capacitor panel, the magnified areas of the board to ITT canon connectors, the surface mounted capacitors, and power connectors, respectively. The insets show the magnified photograph of (C) photoresistors and (D) capacitors employed for operating individual transducer element, respectively. Scale bar represents a length of 50 mm.

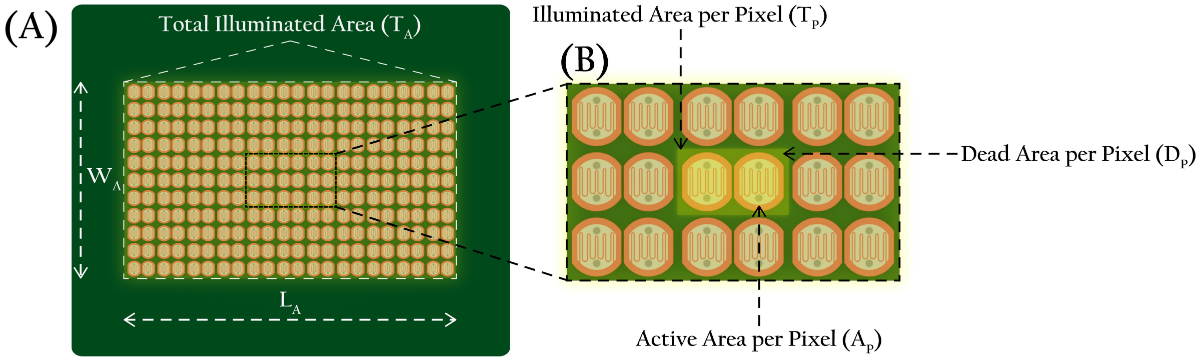

Supplementary Figure S9: **Optical active area in the light-addressable phase shifter.** (A) Total light illuminated area to control the transducer array of 11×11 elements. (B) Magnified schematics of the inset shown with black dotted lines in (A).

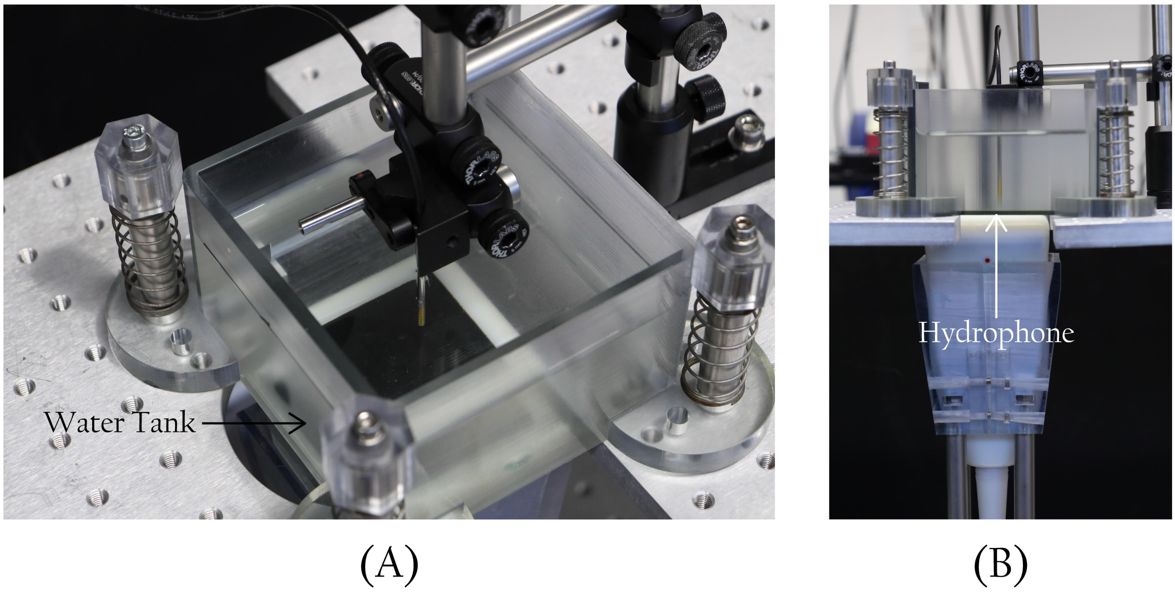

Supplementary Figure S10: **Photographs of the acoustic scanning system to map the acoustic pressure.** The acoustic scanning system includes the hydrophone, custom-mounted transducer array, and custom-built water tank. (A) Top view. (B) Side view.

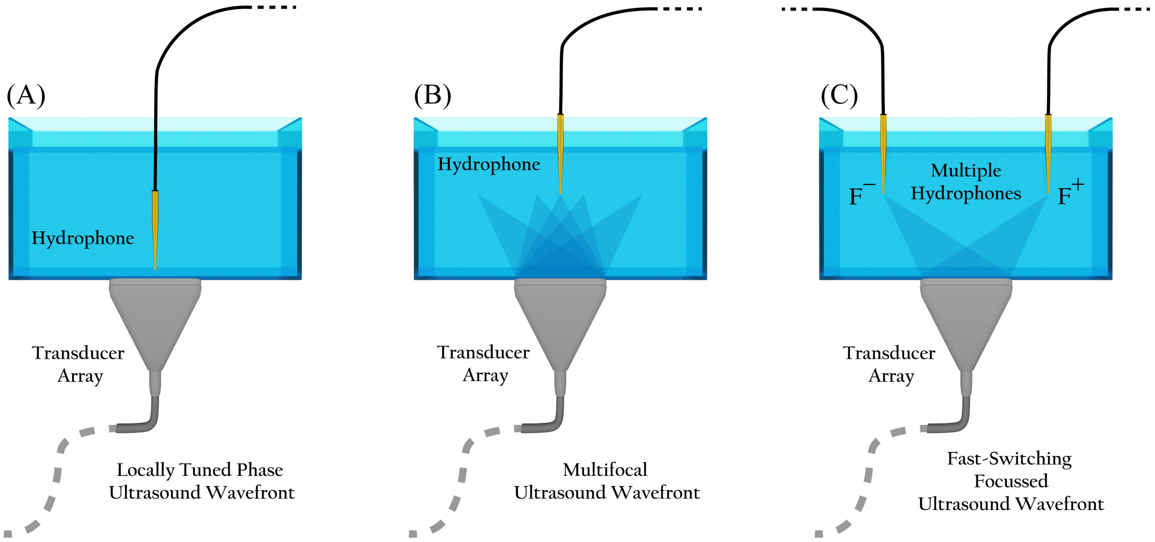

Supplementary Figure S11: **Experimental setups for acoustic pressure measurements.** (A) Determination of phase patterns near the plane of the transducers, the hydrophone is scanned in a plane 1.5 mm from the surface of the transducer array. (B) For the focus and multi-focus measurements, the hydrophone is scanned in a plane 50 mm from the transducer array. (C) The temporal response during switching between the two foci (F+) and (F-), two hydrophones are positioned at the focal points and their output is recorded simultaneously.

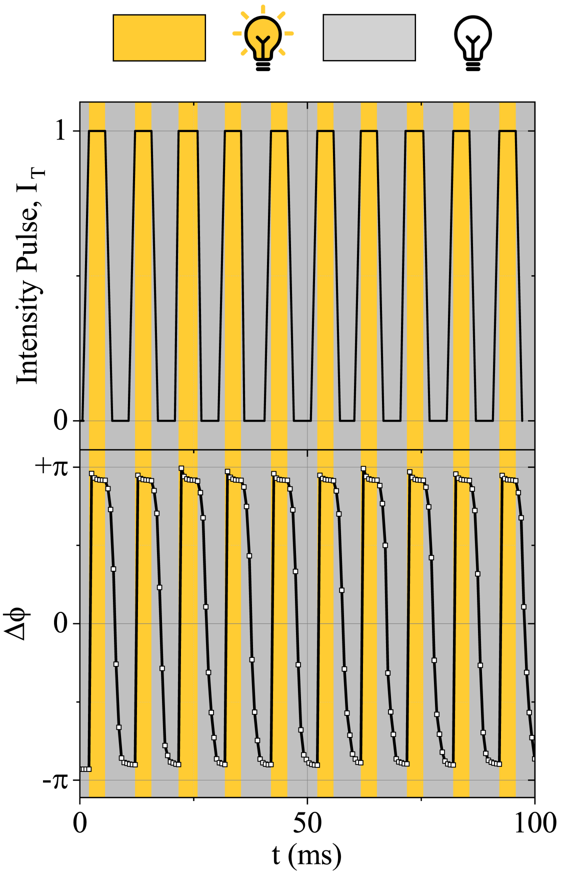

Supplementary Figure S12: **Switching of ultrasound phase at** 100 Hz**.** The phase-shift of the emitted ultrasound wave is measured when the light is switched on ($I_L = 65.7\,\mathrm{mW\,cm^{-2}}$) and off at 100 Hz.

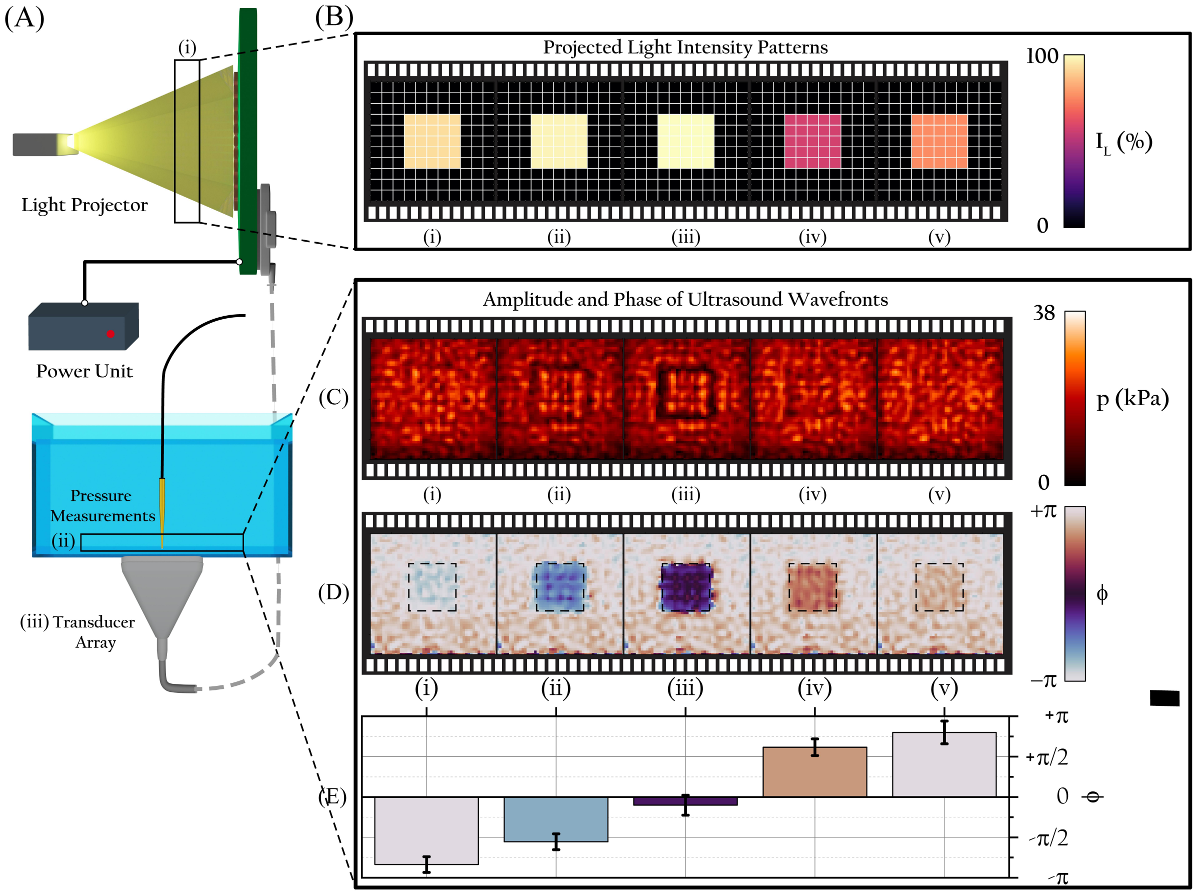

Supplementary Figure S13: **Phase uniformity in the emitted ultrasound wavefront.** (A) Graphical representation of the optically programmable array of transducers. The measurement of the complex amplitude of pressure wave is performed through hydrophone at the close-proximity of the transducer array. The scanning plane is approximately 2 mm from the surface of transducer array. (B) The projected light intensity patterns represented by (A) (i) which are structured as a square shaped with 5×5 sub-array. The maximum projected intensity is 65.7 mW/cm^2. (C) and (D) (i) - (v) The distribution of amplitude and phase-shift in the pressure wave recorded by the hydrophone at the close-proximity ((A) (ii)) of the transducer surface, which corresponds to the projected light patterns of (B) (i) - (v) respectively. (E) The mean and standard deviation of the ultrasound phase plotted in (D) which is constrained in the area enclosed by black dashed line. The approximate area for the scan is 31 mm $\times$ 31 mm. The black color scale bar represents a length of 7.5 mm.

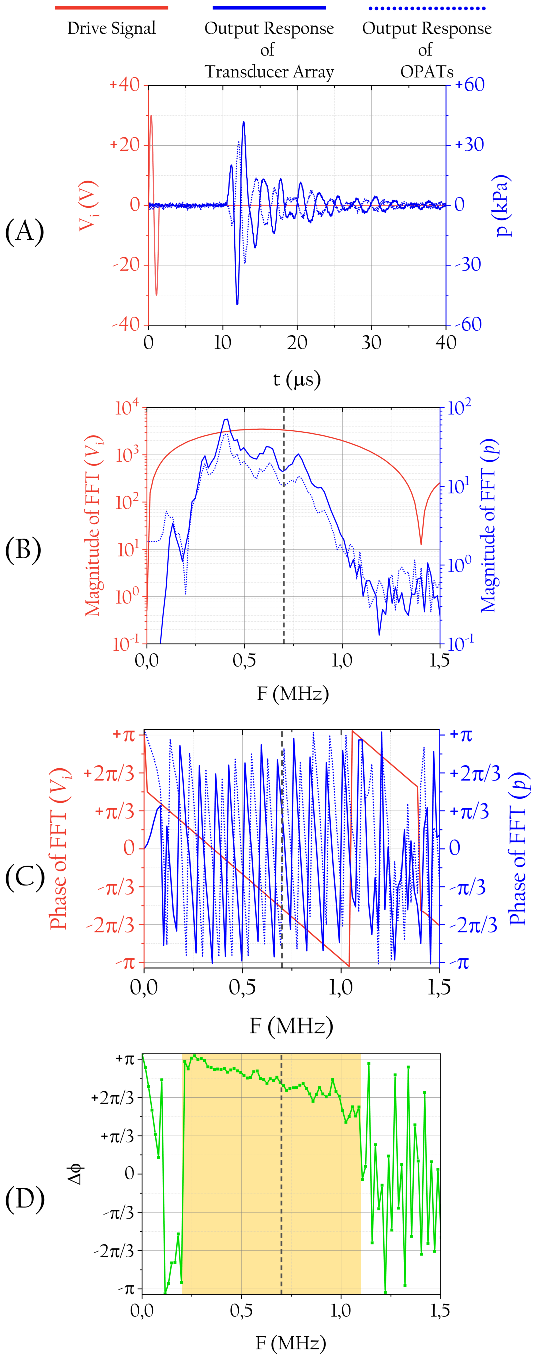

Supplementary Figure S14: **Broadband response of light-addressable transducer array.** An input signal with peak voltage (V_P) of 30 V is applied to the transducer array through direct electrical connection as well as by interfacing it with light addressable electrical architecture. The input voltage signal has a frequency of 0.7 MHz with a time duration of 1.43 µs. (A) The applied drive signal (V_i) and the recorded output pressure waveforms (p) are plotted as a function of time. The Fast Fourier Transforms (FFT) of time-domain signals was estimated, and the magnitude and phase information retrieved from the FFT is presented in (B) and (C) respectively. (D) The phase-shift ($\Delta\phi$) (calculated by subtracting the phases of the output pressure waves corresponding to transducer array and OPATs) is plotted as a function of frequency (F). The yellow color filled area shows the constant phase-shift obtained in a wide-frequency band centered at 0.7 MHz.

REFERENCES

1. Szabo, T. L. *Diagnostic Ultrasound Imaging: Inside Out* ISBN: 9780123964878 (Elsevier, 2014).
2. Lee, J. & Kim, J. Theoretical and Empirical Verification of Electrical Impedance Matching Method for High-Power Transducers. *Electronics* **11,** 194. ISSN: 2079-9292 (2 Jan. 2022).
